# An investigation of the kinetics and thermodynamics of NaCl nucleation through composite clusters

**DOI:** 10.1093/pnasnexus/pgac033

**Published:** 2022-03-30

**Authors:** Pelin S Bulutoglu, Shiyan Wang, Moussa Boukerche, Nandkishor K Nere, David S Corti, Doraiswami Ramkrishna

**Affiliations:** Davidson School of Chemical Engineering, Purdue University, West Lafayette, IN 47907-2100, USA; Davidson School of Chemical Engineering, Purdue University, West Lafayette, IN 47907-2100, USA; Process Research and Development , AbbVie Inc, North Chicago, IL 60064, USA; Process Research and Development , AbbVie Inc, North Chicago, IL 60064, USA; Davidson School of Chemical Engineering, Purdue University, West Lafayette, IN 47907-2100, USA; Davidson School of Chemical Engineering, Purdue University, West Lafayette, IN 47907-2100, USA

## Abstract

Having a good understanding of nucleation is critical for the control of many important processes, such as polymorph selection during crystallization. However, a complete picture of the molecular-level mechanisms of nucleation remains elusive. In this work, we take an in-depth look at the NaCl homogeneous nucleation mechanism through thermodynamics. Distinguished from the classical nucleation theory, we calculate the free energy of nucleation as a function of two nucleus size coordinates: crystalline and amorphous cluster sizes. The free energy surface reveals a thermodynamic preference for a nonclassical mechanism of nucleation through a composite cluster, where the crystalline nucleus is surrounded by an amorphous layer. The thickness of the amorphous layer increases with an increase in supersaturation. The computed free energy landscape agrees well with the composite cluster-free energy model, through which phase specific thermodynamic properties are evaluated. As the supersaturation increases, there is a change in stability of the amorphous phase relative to the solution phase, resulting in a change from one-step to two-step mechanism, seen clearly from the free energy profile along the minimum free energy path crossing the transition curve. By obtaining phase-specific diffusion coefficients, we construct the full mesoscopic model and present a clear roadmap for NaCl nucleation.

Significance Statement Significance: understanding nucleation mechanism for nucleation from solution is crucial for accurate calculation of nucleation rates and for predicting the outcome of crystallization processes. Nevertheless, our understanding is not complete due to limited experimental resolution. This study presents a detailed picture of nucleation mechanism in NaCl nucleation from aqueous solution by utilizing computational tools like molecular simulation, free energy calculations, and a mesoscopic model of nucleation. The analysis presented in the study sheds light onto how the preferred nucleation pathways are affected by supersaturation and can be extended to polymorphic systems for understanding competing polymorph formation pathways.

## Introduction

Crystallization from solution is an important process widely used in the pharmaceutical, chemical, and food industries for separation and purification. Controlling these processes to achieve the desired form of the end product requires a good understanding of the underlying mechanism of nucleation. However, our understanding of nucleation is still far from complete, as a result of the experimental challenges for observing nucleation due to short time and length scales involved in the formation of a critically sized nucleus except for a few systems that are slow enough to be captured by simple microscopy (1,2).

Classical nucleation theory (CNT) offers a simple phenomenological description of nucleation, and has been used widely in theoretical (3–5) and experimental (6–8) studies of nucleation. CNT is based on the assumption that nucleation occurs via a single-step process, where the system overcomes a single free energy barrier and the clusters forming in the solution have the same properties as that of a bulk crystal (9,10). Thus, the size of the emerging nucleus, *n*, is the only slow variable and enough as a reaction coordinate to describe the whole nucleation process. However, nucleation rate predictions of CNT can deviate from experiments by several orders of magnitude (11). Nonclassical nucleation mechanisms have been pointed out as one possible explanation for the discrepancies. In fact, there is evidence for various nonclassical mechanisms like multistep mechanism (12), and nucleation through prenucleation clusters (13,14), composite clusters (15,16), or polycrystalline structures (17). The common feature of these nonclassical mechanisms is that the cluster goes through some structural change as it is emerging from the mother phase and growing to reach the critical size. Thus, some structural order parameter is required in addition to the nucleus size to describe such transformations.

While nucleation is difficult to access experimentally, simulations can provide valuable atomic level insight into the mechanism of nucleation from solution. NaCl nucleation from aqueous solution has been the focus of many computational studies (5,18–29) since it is a simple, well-parameterized system with force fields for which the predicted solubility values are known (30,31). Conclusions on the nucleation mechanism for this system have been varied: large-scale MD simulations done by Chakraborty and Patey (21,22) showed evidence for a two-step mechanism, where nucleation originated in a region of higher salt concentration than that of the bulk solution. They reported early stage nuclei consisting of loosely ordered ions with significant amount of water—inconsistent with the capillarity assumption of CNT. A later study that also employed direct simulations of NaCl nucleation (23) revealed that the expected lifetime and nucleation probability of clusters depended not only on their size but also the geometric arrangement of their ions. Zimmerman et al. used seeded simulations to estimate the nucleation rates at 3 different supersaturations and were able to match the trend of experimental measurements. It is worth noting that the seeding approach uses elements from CNT for calculating the nucleation rate, inherently assuming a single-step nucleation mechanism (32). A later study (25) showed that the chemical potential of NaCl ions in solution increased as a function of concentration, until it reached a plateau after a concentration of 15.0 mol/kg.This plateau is explained with a spinodal decomposition that leads to a barrierless, spontaneous formation of amorphous clusters, which is the first step of the nucleation process. Then, crystallization within the amorphous clusters proceeds via a finite barrier, which constitutes the second step.

In this study, we examine the mechanism of NaCl nucleation from solution and provide a complete thermodynamic picture that reveals the preferred pathway of nucleation. As pointed out by earlier studies (23), the degree of order of the clusters affect their lifetime and nucleation probability. Therefore, a single dimensional description of nucleation with a nucleus size reaction coordinate is not enough to capture the structural changes in the cluster. In addition to the crystalline nucleus size reaction coordinate, we introduce a second nucleus size variable to monitor the formation of the amorphous phase as well as the crystalline phase and show the preferred pathway of nucleation through free energy calculations. Furthermore, using a model based on previous literature (16), we study the full thermodynamic picture based on the 2D free energy surface from the molecular simulations. We then calculate the nucleation rate from the proposed mesoscopic system.

## Materials and Methods

### Setup of molecular simulations

We studied nucleation of NaCl from aqueous solution at two concentrations: 15.0 and 18.0 mol/kg. The NaCl interactions were calculated using the Joung–Cheatham (JC) force field (33) and the SCP/E water model (34) was used for the water molecules. The predicted NaCl solubility in water for the chosen force field is 3.7 mol/kg (30), which corresponds to a supersaturation of 4.05 and 4.8 at the chosen concentrations. The free energy calculations were done with 500 NaCl molecules and 1,851 water molecules.

All molecular dynamics (MD) simulations were done with LAMMPS Molecular Dynamics Simulator (35). Periodic boundary conditions were implemented in all directions. The simulations were run at 298 K and 1 atm. Temperature and pressure couplings were done using the Nose–Hoover thermostat (36) with a time constant of 100 time steps and Nose–Hoover barostat with a time constant of 1,000 time steps, respectively. A cut-off value of 9.0 Å; was used for the Lennard–Jones and Coulombic interactions. Long range Coulombic interactions were calculated with the particle-particle-particle-mesh (pppm) solver with an accuracy of 10^−4^.

### Mesoscopic system

#### Selection of reaction coordinates

To monitor the formation of clusters and their structural changes, we use two collective variables: the number of ions in the largest dense cluster (*n*_ρ_) and the number of ions the largest crystalline cluster (*n_c_*).

The strategy for calculating the cluster sizes was inherited from Jiang et al. (25): The dense cluster size was calculated by first calculating the local density for each ion in the system. The local density was defined as the number of neighbors (ρ) an ion has within a cut-off radius *r_cut_* of 0.45 nm. An ion *i* was identified as ”solid-like” if ρ^(*i*)^ > 8. The choice of 8 for the number density cut-off was chosen based on previous literature (25), but the number density distribution of ions in a system with a crystalline slab embedded in aqueous solution supports the choice of this cut-off (see Figure S3, Supplementary Material). A clustering algorithm was used to cluster the solid-like ions, assuming that two solid-like ions belong to the same cluster if they are within 0.45 nm of each other. The *freud* library (37) was used to evaluate the number of neighbors and for clustering. The number of ions in the largest cluster identified with the described method was taken as *n*_ρ_. The degree of crystallinity of the ions was not considered in calculating this variable; thus, it does not carry any information about the structure of the cluster.

For calculating the crystalline cluster, the ions were first evaluated for their crystallinity by calculating their Steinhardt bond-orientational order parameter (38), denoted as *q*_8_. The order parameter for the *i*th ion was calculated as follows (23,25):

(1) $$\begin{equation*}
q_8{(i)} = \sqrt{\sum _{m=-8}^{8} |q_{8m}(i)|^2},
\end{equation*}
$$

where *q*_8*m*_(*i*) is given as

(2) $$\begin{equation*}
q_{8m}(i) = \frac{1}{N_B} \sum _{j=1}^{N_B} Y_{8m}(\theta (\mathbf {r}_{ij}), \phi (\mathbf {r}_{ij})).
\end{equation*}
$$

*Y*_8*m*_ is the *m*th component of the 8th order spherical harmonics function between ion *i* and its neighbor *j*. The spherical harmonics functions were averaged over the *N_B_* = 12 nearest neighbors of the *i*th ion. An ion *i* was considered to be crystalline if *q*_8_(*i*) is greater than a threshold value of 0.45. Again, the crystalline ions were clustered assuming that two crystalline ions are connected if they are within 0.35 nm of each other. The number of ions in the largest crystalline cluster identified with the described method was taken as *n_c_*.

**Fig. 1:**
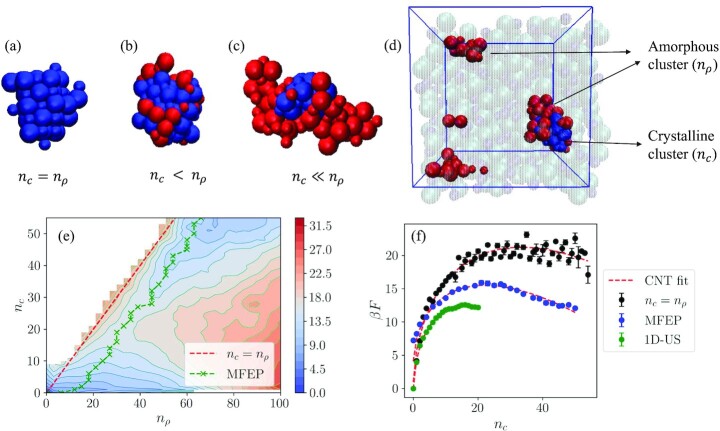
(a) A purely crystalline cluster for which *n_c_* = *n*_ρ_. (b) A mostly crystalline cluster with a few amorphous particles around, and (c) a composite cluster composed of crystalline particles surrounded by an amorphous layer, for which *n_c_* < *n*_ρ_. Particles that are identified as crystalline are shown in blue, whereas particles that are identified as ”solid-like” but not crystalline are shown in red. (d) A configuration taken from an MD simulation at *m* = 15.0 mol/kg. (e) Contour plot of β*F*(*n_c_*, *n*_ρ_). The red dashed line is the *n_c_* = *n*_ρ_ line, which corresponds to the single-step nucleation pathway. The green crosses correspond to the MFEP. (f) Free energy profile plotted against *n_c_* along the *n_c_* = *n*_ρ_ line and the MFEP. The free energy barrier obtained from 1D umbrella sampling simulations is included in green for comparison. Red dashed lines are CNT (Eq. 19) fits into the free energy data. Error bars are calculated from block averaging with 5 blocks.

It is worth noting that, since *n*_ρ_ is calculated based only on the local density of the particles, it includes all dense particles that are crystalline as well as amorphous. Thus, for a purely crystalline particle, *n_c_* will be equal to *n*_ρ_. For a composite cluster, which is composed of crystalline particles surrounded by an amorphous layer, *n_c_* will be less than *n*_ρ_ as shown in Fig. 1(a)–(c). Finally, *n_c_* can never exceed *n*_ρ_ since the crystalline particles will always be dense. The choice of cut-off values and neighbor distance criteria when calculating the order parameters can result in over/underestimation of cluster sizes. This will inevitably affect our quantitative results, especially the values of the thermodynamic parameters that are obtained from fitting the composite cluster model to the free energy surface. However, a quick analysis detailed in Figures S4 and S5 (Supplementary Material) showed that the qualitative conclusions of the study are not sensitive to the number density cut-off value.

#### Free energy calculation

The 2D free energy surface as a function of the dense and crystalline nucleus sizes, $\beta F(\mathbf {n})$, where $\mathbf {n} = (n_{\rho },n_c)$, was obtained by running 2D umbrella sampling (39) simulations with hybrid Monte Carlo/Molecular Dynamics (HMC/MD) (40). Details of the HMC/MD method can be found in the Supplementary Material along with the validation of the method in Figure S1 (Supplementary Material). The free energy is computed from

(3) $$\begin{equation*}
\beta F(\mathbf {n}) = -\log \left[ P(\mathbf {n})\right] + C,
\end{equation*}
$$

where $P(\mathbf {n})$ is the joint probability of observing a system with largest nucleus sizes of *n_c_* and *n*_ρ_. The value of the constant *C* is chosen so that β*F*(0, 0) is 0. The joint probability is estimated from 2D umbrella sampling simulations, using a biasing potential

(4) $$\begin{equation*}
U_B(\mathbf {n}; \mathbf {K}, \mathbf {n}^{*}) = \frac{1}{2}(\mathbf {n}-\mathbf {n}^{*})^{\top } \mathbf {K} (\mathbf {n} - \mathbf {n}^{*}),
\end{equation*}
$$

where $\mathbf {K}$ is a 2 × 2 diagonal matrix containing the biasing constants in each dimension and $\mathbf {n}^{*}$ is the vector of target values to be sampled in a given simulation for *n_c_* and *n*_ρ_. It should be noted that the Landau free energy in Eq. 3 based on the largest cluster size in the system does not agree with CNT at small cluster sizes, since CNT predicts the reversible work of formation of a cluster of size *n*, and hence, is related to the population of nuclei of size *n*. The two definitions are approximately equal to each other at large clusters since it is unlikely to find two large clusters in the system (41). See the text and Figure S2 (Supplementary Material) for more detail about free energy calculations. The biasing constants were chosen as 0.15 kcal/mol and 0.07 kcal/mol for *n_c_* and *n*_ρ_, respectively, which allowed the average acceptance ratio of the MC moves to stay near 20%. The target values were varied between 0 and 50 for *n_c_* and 0 and 100 for *n*_ρ_, with enough windows to ensure that there is good overlap between samples from each window. A total of 80,000 HMC steps were collected from each umbrella window and the first 30,000 were discarded to ensure equilibrium sampling. The joint probabilities obtained from each umbrella window were combined using WHAM (42).

#### Mesoscopic model

The Fokker–Planck equation in multidimensional collective variable space is written as

(5) $$\begin{equation*}
\frac{\partial \rho (\mathbf {n},t)}{\partial t}=-\nabla \cdot (\boldsymbol {\mu }(\mathbf {n})\rho -\nabla \cdot (\mathbf {D}(\mathbf {n})\rho )),
\end{equation*}
$$

where $\boldsymbol {\mu }$ is the drift term, $\mathbf {D}$ is the diffusivity tensor, and $\rho (\mathbf {n},t)$ is the probability density with $\mathbf {n}$ representing phase space variables. Under equilibrium conditions, the probability flux is zero, therefore ,

(6) $$\begin{eqnarray*}
\mu (\mathbf {n})\rho _{eq}(\mathbf {n})& = &\nabla \cdot (\mathbf {D}\rho _{eq}(\mathbf {n})),
\end{eqnarray*}
$$

(7) $$\begin{eqnarray*}
\mu (\mathbf {n})& = &\nabla \cdot \mathbf {D} - \beta \mathbf {D}\cdot \nabla F(\mathbf {n}).
\end{eqnarray*}
$$

In going from Eqs. 6 to 7, we use the fact that $\rho _{eq}~= e^{-\beta F(\mathbf {n})}$, where $F(\mathbf {n})$ is the free energy as a function of the nucleus size variables and β is 1/*kT*, the inverse of thermal energy. Assuming constant diffusion coefficient and substituting Eq. 7 into Eq. 5, we obtain

(8) $$\begin{equation*}
\frac{\partial \rho (\mathbf {n},t)}{\partial t}=\nabla \cdot \left[e^{-\beta F(\mathbf {n})}\mathbf {D}\cdot \nabla \left(e^{\beta F(\mathbf {n})}\rho (\mathbf {n},t)\right)\right].
\end{equation*}
$$

The rate of crossing between two states can be obtained from the steady-state nonequilibrium flux of trajectories over the barrier (43,44). If the barrier is large enough, it can be assumed that the reactant side of the barrier will quickly reach equilibrium. If each trajectory that escapes to the product side is removed and replaced on the reactant side, the system will reach a nonequilibrium steady state. The steady state probability distribution of the system should satisfy the steady state Fokker–Planck equation:

(9) $$\begin{equation*}
\nabla \cdot \left[ e^{-\beta F(\mathbf {n})} \mathbf {D} \cdot \nabla \Phi \right] = 0,
\end{equation*}
$$

where $\Phi \equiv \rho (\mathbf {n})/\rho _{eq}(\mathbf {n})$, known as Kramer’s crossover function (45). To compute the nucleation rate constant, *I*, one should solve Eq. 9 and evaluate the total probability flux through a dividing surface between the solution state and the state with the nucleated cluster:

(10) $$\begin{eqnarray*}
I = \int _S \boldsymbol {\nu } \cdot \mathbf {J} dS,
\end{eqnarray*}
$$

where $\mathbf {J}=-\rho _{eq}(\mathbf {n})\mathbf {D}\cdot \nabla \Phi$ is the probability flux, *S* is a dividing hyper surface in the collective variable space, and $\boldsymbol {\nu }$ is the normal to that surface.

The diffusivity tensor $\mathbf {D}$ (*D_ij_* as index notation) is evaluated by estimating the mean squared deviation (MSD) of the collective variables, $\mathbf {n} = [n_{\rho }, n_c]$, from short MD trajectories and using the Einstein relation (46–48)

(11) $$\begin{equation*}
D_{ij} = \frac{\text{MSD}_{ij}(\tau )}{2\tau },
\end{equation*}
$$

and

(12) $$\begin{eqnarray*}
\text{MSD}_{ij}(\tau ;t_f) &=& \frac{1}{t_f-\tau } \int _0^{t_f-\tau } \langle \left[ \delta n_i(t^{\prime }+\tau ) -\delta n_i(t^{\prime }) \right] \nonumber \\
&&\times \left[ \delta n_j(t^{\prime }+\tau ) -\delta n_j(t^{\prime }) \right] \rangle dt^{\prime },
\end{eqnarray*}
$$

where δ*n_i_*(*t*) = *n_i_*(*t*) − 〈*n_i_*(*t*)〉 and *t_f_* is the observed length of the trajectories. It should be noted that systematic drift was subtracted from the trajectories before they are used in the calculation of MSDs. The systematic drift, 〈*n_i_*(*t*)〉, was evaluated by starting four independent trajectories from the same initial configuration with initialized velocities and averaging over the trajectories. This is done to prevent the contribution of any systematic drift to diffusion coefficient calculations (49). The deviations are averaged over both time and independent trajectories for improved statistics.

## Results and Discussion

### NaCl nucleation: minimum free energy path

Figure 1(e)–(f) shows the 2D free energy surface obtained at the same concentration (15.0 mol/kg), now as a function of both the crystalline and dense clusters. The red dashed line in Fig. 1(e) is the *n_c_* = *n*_ρ_ line. This line corresponds to the classical mechanism, since the cluster is purely crystalline along the line and there is no intermediate amorphous phase. The other pathway displayed on Fig. 1(e) is the minimum free energy path (MFEP) on the free energy surface, which is the path that the system is free energetically most favored to follow (50). It is apparent that the dense cluster size is larger than the crystalline nucleus size at all points along the MFEP, indicating the presence of an amorphous layer around the crystalline cluster throughout the nucleation pathway.

**Fig. 2:**
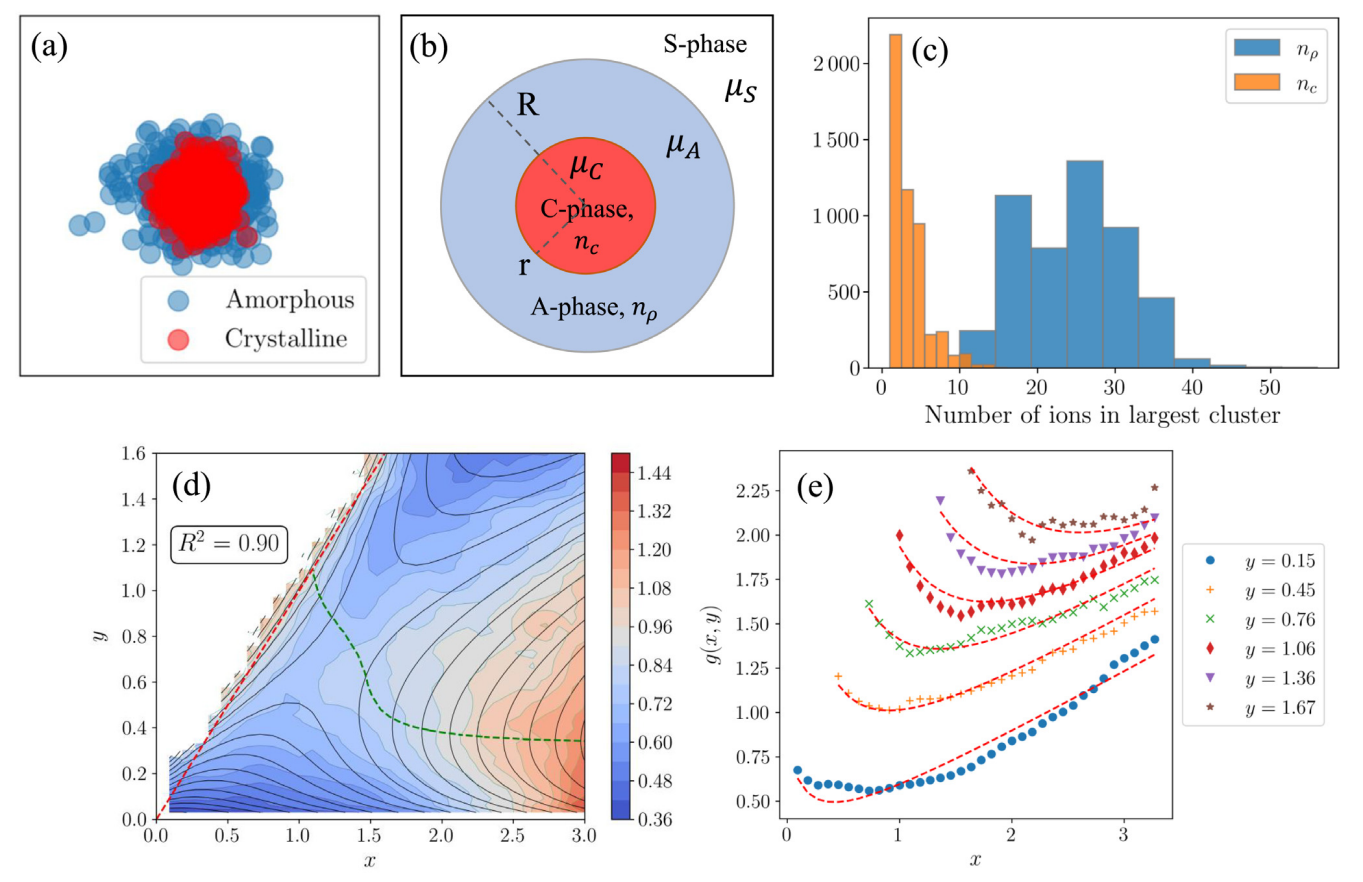
(a) Superposition of 20 cluster configurations collected near the saddle point. The 3D configurations of the clusters are projected onto the x–y plane for visualization purposes. (b) Schematic of the composite cluster. A: amorphous, C: crystalline, and S: solution. (c) Distribution of sizes of the largest dense and crystalline clusters in a MD simulation of aqueous NaCl solution at 15.0 mol/kg. (d) Fit of Eq. 22 onto the 2D free energy surface. The black contour lines are the fit, the colored surface plot is the data obtained from umbrella sampling simulations. The green dashed line shows the transition curve, explained in the text. (e) Fixed *y* lines of the free energy surface, plotted against the dimensionless dense nucleus size, *x*. Red dashed lines correspond to Eq. 22, markers are the umbrella sampling data. The free energy curves for *y* > 0.15 are shifted up along the *y*-axis for ease of viewing.

Figure 1(f) shows the change in free energy along the MFEP, in comparison with the free energy along the *n_c_* = *n*_ρ_ line as well as the free energy profile obtained from the 1D umbrella sampling simulations. The MFEP includes the saddle point, which is located at *n_c_* = 22 and *n*_ρ_ = 35. The comparison shows that the classical pathway requires the system to cross a higher free energy barrier compared to the MFEP, which indicates that the nonclassical pathway is thermodynamically more favored than the classical pathway at the concentration of 15.0 mol/kg. It should be noted that the MFEP still includes a single barrier, as opposed to what is expected in a two-step nucleation scenario (51,52). Therefore, we conclude that the mechanism of nucleation at this level of supersaturation is not a two-step mechanism but not necessarily classical as well. Rather, nucleation takes place with a nonclassical mechanism through the formation of composite clusters made up of crystalline and amorphous particles, namely, a composite cluster pathway. The superposition of 20 cluster configurations collected near the saddle point is shown in Fig. 2(a), which shows that the amorphous particles tend to form a layer around the crystalline cluster at the center of the cluster, in accordance with the composite cluster model.

### The composite cluster pathway

The free energy change of forming a composite cluster has been formulated for nucleation of a solid from the gas phase in the presence of an intermediate liquid phase using capillarity approximation (16). The vapor–liquid–solid nucleation can be considered analogous to nucleation of a crystalline cluster from solution in the presence of an intermediate amorphous phase. To be able to compare with the simulation results, the model is reformulated here to obtain a free energy expression in terms of number of particles in the nucleus, instead of cluster radii. A schematic of the composite cluster is given in Fig. 2(b).

The three phases considered in the system are the solution phase (S), amorphous phase (A), and the crystalline phase (C). The free energy change of formation of a composite cluster in solution is the sum of the individual contributions:

(13) $$\begin{equation*}
\Delta F = \Delta F_{AS} + \Delta F_{CA} + \Delta F_{CAS},
\end{equation*}
$$

where Δ*F_AS_* is the free energy of formation of the amorphous cluster in solution and Δ*F_CA_* is the free energy of formation of a crystalline cluster within the amorphous phase. Based on the capillarity approximation, Δ*F_AS_* and Δ*F_CA_* can be expressed in terms of cluster sizes as

(14) $$\begin{eqnarray*}
\Delta F_{AS} = -\Delta \mu _{AS}n_{\rho } + 4\pi R^2 \sigma _{AS},
\end{eqnarray*}
$$

(15) $$\begin{eqnarray*}
\Delta F_{CA} = -\Delta \mu _{CA}n_c + 4\pi r^2 \sigma _{CA},
\end{eqnarray*}
$$

where Δμ_*AS*_ = μ_*S*_ − μ_*A*_, the difference in the chemical potentials of the solution and amorphous phases. *R* and *r* are the radii of the composite and crystalline clusters, respectively, and σ_*ij*_ is the surface energy of the interface between phases i and j. Δ*F_CAS_* is a correction term to account for the short-range interaction between the C–A and A–S interfaces (16), given as

(16) $$\begin{equation*}
\Delta F_{CAS} = 4\pi R^2 S e^{-(R-r)/\xi },
\end{equation*}
$$

where ξ is a parameter that specifies the range of interaction, and *S* is the spreading parameter:

(17) $$\begin{equation*}
S=\sigma _{CS}-\sigma _{AS} - \sigma _{CA}.
\end{equation*}
$$

Equations 14–17 are combined to obtain the free energy expression

(18) $$\begin{eqnarray*}
\Delta F &=& -n_{\rho }\Delta \mu _{AS} - n_c \Delta \mu _{CA} \nonumber \\
&&+\,\, 4\pi R^2 \left[ \sigma _{AS} + \sigma _{CA} \left( \frac{r}{R}\right)^2 + S e^{-(R-r)/\xi } \right] .
\end{eqnarray*}
$$

Setting *n_c_* = *n*_ρ_ (and *R* = *r*), Eq. 18 reduces to the CNT expression for the free energy change of single-step nucleation of a crystalline cluster from solution:

(19) $$\begin{equation*}
\Delta F_{CS} = \Delta \mu _{CS} n_c + 4\pi r^2 \sigma _{CS},
\end{equation*}
$$

from which the critical nucleus size and free energy barriers can be obtained assuming spherical geometry as

(20) $$\begin{equation*}
\Delta F^{*}_{CS} = \frac{16\pi }{3}\frac{\sigma ^3_{CS}}{\Delta \mu _{CS}^2}\nu _m^2, \qquad N_{CS}^{*} = \frac{32 \pi }{3} \left( \frac{\sigma _{CS}}{\Delta \mu _{CS}}\right)^3 \nu _m^2,
\end{equation*}
$$

where ν_*m*_ is the molecular volume, assumed to be identical for the crystalline and amorphous phases. Using the dimensionless parameters from Eq. 16:

(21) $$\begin{eqnarray*}
x &=& \frac{n_{\rho }}{N_{CS}^{*}}, \quad y = \frac{n_c}{N_{CS}^{*}}, \quad \alpha = \frac{\sigma _{CA}}{\sigma _{CS}}, \quad \beta = \frac{\sigma _{AS}}{\sigma _{CS}}, \quad S^{\prime } = \frac{S}{\sigma _{CS}}\nonumber\\
\delta &=& \frac{\Delta \mu _{AS}}{\Delta \mu _{CS}}, \quad \tau = \frac{\xi }{(\frac{3}{4\pi } N_{CS}^{*} \nu _m)^{1/3}}, \quad g(x,y) = \frac{\Delta F (n_{\rho }, n_c)}{\Delta F_{CS}^{*}},
\end{eqnarray*}
$$

Equation 18 is nondimensionalized:

(22) $$\begin{eqnarray*}
g(x,y) &=& -2\delta x - 2(1-\delta )y + 3x^{2/3} \nonumber \\
&&\times \left[\beta + \alpha \left(\frac{y}{x} \right)^{2/3} + S^{\prime } \exp {\left(-\frac{x^{1/3}-y^{1/3}}{\tau }\right)} \right],
\end{eqnarray*}
$$

where

(23) $$\begin{equation*}
S^{\prime } = 1-(\beta + \alpha ).
\end{equation*}
$$

Numerical values for $\Delta F_{CS}^{*}$ and $N_{CS}^{*}$ were obtained from fitting Eq. 19 into the free energy profile along the *n_c_* = *n*_ρ_ line, shown in Fig. 1(f). The resulting parameters from the fit are shown in Table 1. The interfacial energy was determined from Eq. 20, taking ν_*m*_ as the reciprocal of the number density of a NaCl crystal simulated with the JC force field (0.024 nm^3^/ion). The free energy surface obtained from simulations was scaled using the estimated $\Delta F_{CS}^{*}$ and $N_{CS}^{*}$ values and the nondimensional free energy expression in Eq. 22 was fitted to the scaled free energy surface. The nondimensional parameters α, β, δ, and τ are obtained from parameter estimation using nonlinear least squares.

**Table 1.** Parameters obtained from fitting the CNT expression (Eq. 19) into the free energy profile along the *n_c_* = *n*_ρ_ pathway.

Parameters	*m* = 15 mol/kg	*m* = 18 mol/kg
$\beta \Delta F_{CS}^{*}$	21.2 ±1.6	21.3 ±3.0
$N_{CS}^{*}$	33 ±10	20 ±7
Δμ_*CS*_ (kJ/mol_ion_)^a^	3.03 ±0.3	5.9 ±0.4
σ_*CS*_ (mJ/m^2^)	60.05 ±2.8	98.4 ±5.7

^a^Note that this value is given per mol of ion and should be doubled to obtain the chemical potential difference per mol of NaCl molecule.

Figure 2(d) shows the resulting fit in comparison with the simulation results. It is observed that the composite cluster model agrees well with the free energy surface obtained from simulations, as the *R*^2^ value of 0.9 suggests. The agreement between the fit and the data can be observed clearly in Fig. 2(e), where constant *y* lines of *g*(*x, y*) are plotted against *x* for both Eq. 22 and the simulation data. This shows that the nucleation mechanism for NaCl at the chosen conditions can be described well by the composite cluster model. It is worth noting that the cluster sizes involved in the simulations are between 0 and 100 ions, which corresponds to cluster radii less than 1 nm^3^. At these cluster sizes, it may be expected that the capillarity approximation does not hold. The shape of the clusters is also an important factor, which is assumed to be spherical in the model. It is unrealistic to expect perfectly spherical clusters for the small sizes that this study focuses on. The sphericity analysis based on the moment of inertia tensors of the clusters showed that the clusters along the MFEP are never fully spherical but their sphericity increase as they grow, as well as their crystallinity (see Figures S6 and S7, Supplementary Material). Nevertheless, Iwamatsu’s capillarity approximation-based model still agrees well with the simulation results at small cluster sizes.

### Thermodynamics of nucleation pathways

Also shown in Fig. 2(d) is the transition curve. The transition curve can be defined as the collection of points located on the ridge-line between the two states, as described by Voter (53). In contrast to the 1D representation of the system where the transition state is defined by a single point, we now have a collection of nucleus sizes through which the system can cross over to the nucleated state. The transition curve gives us an idea on how the critical crystalline cluster size changes with the size of the largest dense cluster in the system. It is observed that smaller crystalline clusters are enough to initiate nucleation in the presence of larger dense clusters. The fact that the critical crystalline cluster size changes along the transition curve is in agreement with the results of Lechner *et al*. (54), where they employed a likelihood maximization technique to identify the order parameters that are important for homogeneous crystal nucleation in a soft core colloid model. Their results showed that information about the size of the disordered surface cloud as well as the crystalline nucleus size is necessary to predict the fate of a given nucleus.

The thermodynamic parameters obtained from the fit are shown in Table 2. The negative δ value indicates that the chemical potential difference between the amorphous and the solid phase is negative. This means that the amorphous phase is less stable than the solution phase. However, σ_*AS*_, which is the surface energy of the amorphous–solution interface, is also predicted to be negative. Although unusual, negative surface free energy was recognized by Tolman (55) and reported for droplets in supercooled LJ vapor by Corti *et al*. (56). Surface energy dominates the thermodynamics at small cluster sizes and formation of small amorphous clusters becomes thermodynamically favorable even though the amorphous phase is less stable than the solution phase. This explains the instantaneous formation of small amorphous clusters in solution, which is observed in MD simulations of NaCl solution at 15.0 mol/kg concentration as shown in Fig. 2(c). It can be concluded that, at the concentration of 15.0 mol/kg NaCl solution, the amorphous phase is the least stable phase, followed by the solution phase and the crystalline phase, which is the most stable.

**Table 2.** Thermodynamic variables obtained through parameter estimation by fitting Eq. 22 into the simulation results at the two supersaturations studied.

Parameters	*m* = 15 mol/kg	*m* = 18 mol/kg
σ_*CA*_ (mJ/m^2^)	50.0 ± 2.4	167 ± 10
σ_*AS*_ (mJ/m^2^)	−3.2 ± 0.5	103 ± 7
Δμ_*AS*_ (kJ/mol_ion_)	−0.63 ± 0.07	3.5 ± 0.3
ξ (nm)	0.079 ± 0.008	0.42 ± 0.05

### Nucleation rate

**Fig. 3:**
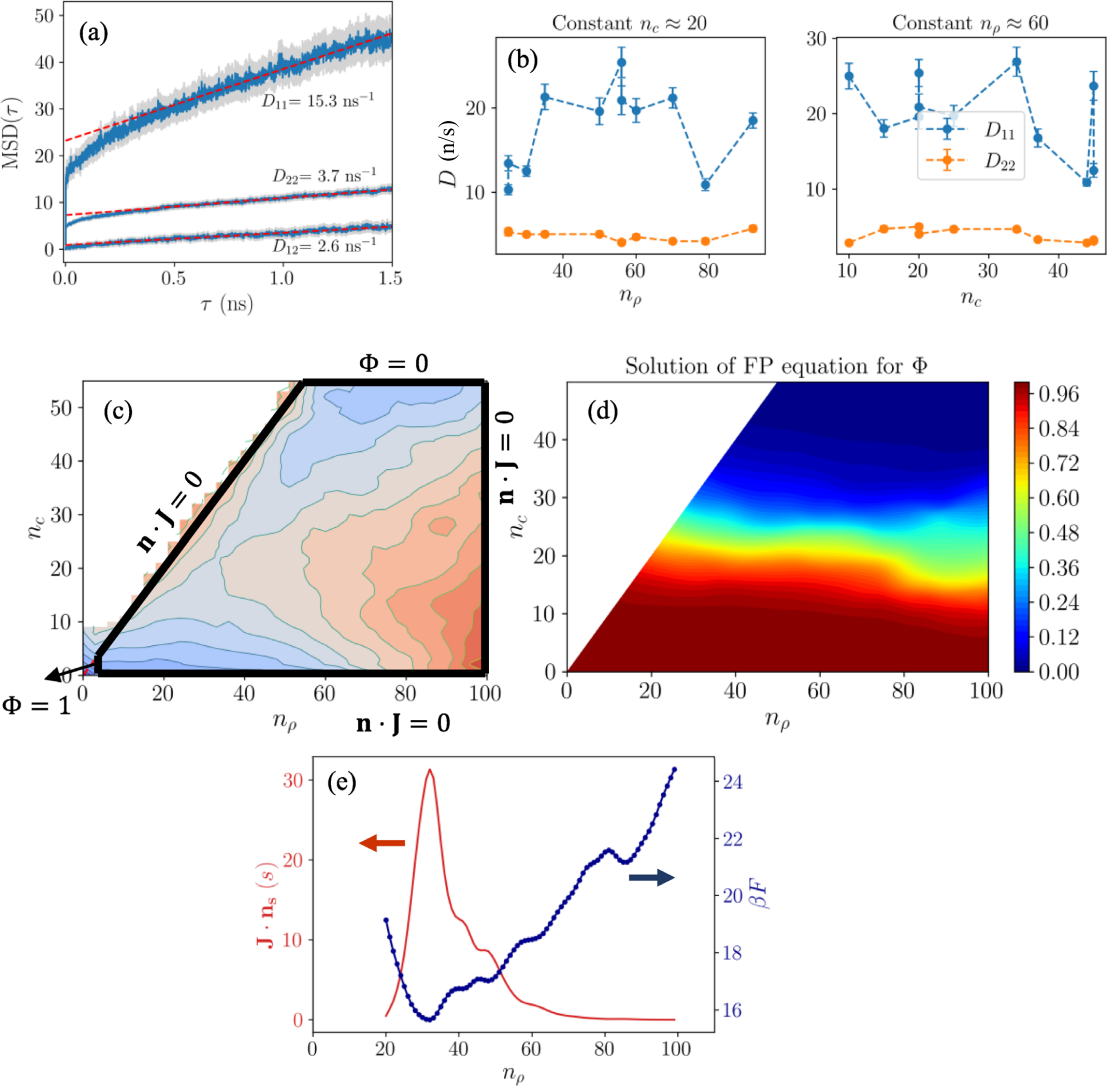
(a) MSD profiles calculated from trajectories starting at the saddle point (*n_c_* = 22 and *n*_ρ_ = 35). MSD profiles are averaged in time and over 100 independent trajectories. Red dashed lines are the linear fits on the MSD curves using data between 0.5 and 1.50 ns. (b) Dependence of the estimated diffusion coefficients of the *n*_ρ_ (*D*_11_) and *n_c_* (*D*_22_) size variables on nucleus size. (a) The domain used for solving Eq. 9 and the boundary conditions. No flux boundary conditions are employed for the right and bottom boundaries as well as the *n*_ρ_ = *n_c_* line. Near the solution state, where *n*_ρ_ = 1, Φ is taken as 1 in accordance with the quasi-steady state assumption. (b) Solution of Eq. 9. The nucleation rate is obtained by evaluating the total flux through the top boundary, *n_c_* = 50. (c) Free energy profile (shown in blue on the right axis) and the magnitude of the probability flux (shown in red on the left axis) through each point on the transition curve.

To see whether inclusion of the *n*_ρ_ coordinate has an effect on the nucleation rate, we calculated the nucleation rate from the 2D system by solving Eq. 9. The diffusivity tensor that is needed for solving Eq. 9 is evaluated on the saddle point using direct MD simulations and calculating the MSD using Eqs. 11 and 12. Independent simulations were started from 25 chosen configurations that are close to the saddle point in the collective variable space. From each configuration, 4 different trajectories were started by initializing the velocities, amounting to a sum of 100 independent trajectories per point. This is done to remove any possible drift (5) as explained earlier. The resulting MSD curves and the extracted diffusivities are shown in Fig. 3(a). The components of the diffusivity tensor are estimated to be *D*_11_ = 15.3 ± 0.9, *D*_22_ = 3.7 ± 0.2, and *D*_12_ = 2.6 ± 0.3 n/s. Here, the first component is the size of the dense cluster and the second component is the size of the crystalline cluster. The diffusion coefficient of the crystalline cluster is slightly less than the calculated value by Jiang et al. (25) at the same supersaturation, reported as 4.6 n/s. It is observed that the diffusion coefficient of the dense cluster is about 4 times that of the crystalline cluster, which indicates that ions can attach to the dense cluster easier compared to the crystalline cluster. This is likely because the ions need to both diffuse to the surface of the cluster and reorganize to be part of the crystalline cluster, whereas this reorganization is not needed to attach to the amorphous layer of the dense cluster.

As mentioned earlier, we assume the diffusivity to be independent of the cluster size and use the diffusion coefficients evaluated on the saddle point. This assumption is based on evaluations of diffusion coefficients at various points on the collective variable space, as shown in Fig. 3(b). It is seen that as the diffusivity of the crystalline cluster remains constant with size, there are big fluctuations in the diffusivity of the dense cluster, with no clear trend of increase or decrease with increasing size. With known free energy and diffusivity terms, Eq. 9 can now be solved to obtain the nucleation rate. Finite difference was used for numerically solving the equation with a step size of *h* = 0.03. Since the free energy surface is known only for discrete values of the collective variables (and the nucleus sizes themselves are discrete variables), values in between were obtained using interpolation with spline fitting. The chosen boundary conditions are shown in Fig. 3(c) and the solution for the crossover function Φ is shown in Fig. 3(d).

The nucleation rate was obtained by evaluating the flux and using Eq. 10 as 4.3 ± 3.9 × 10^3^/s. This value is in agreement with those reported in the literature at *m* = 15.0 mol/kg by both brute force simulations and by using CNT rate expression (25). The agreement with CNT is remarkable, since our analysis showed that the nucleation mechanism is nonclassical at this supersaturation. There may be multiple reasons for this agreement: Although the nucleation mechanism is shown to be nonclassical in the sense that nucleation occurs through the formation of composite clusters, it is still a single-step process where the system needs to overcome a single activation barrier for nucleation. Moreover, analysis of the magnitude of the probability flux along the transition curve (Fig. 3e) shows that the majority of the probability flux occurs through a narrow distribution of nucleus sizes. This is in accordance with the classical theory that considers a single point as the transition state.

### Higher supersaturation case

**Fig. 4:**
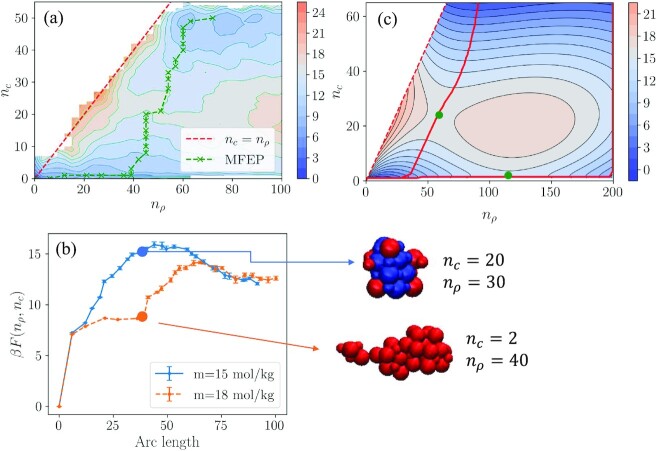
(a) Free energy surface for nucleation at *m* = 18.0 mol/kg. The green crosses show the MFEP. (b) Comparison of the free energy profile along the arc length of the MFEP at *m* = 15.0 mol/kg (blue solid line) and *m* = 18.0 mol/kg (orange dashed line). Also shown are snapshots of clusters collected from the same arc length on the MFEPs at *m* = 15 and 18 mol/kg. Particles marked as amorphous are shown in red, whereas the crystalline particles are shown in blue. (c) Extrapolation of the free energy surface to larger nucleus sizes using Eq. 22 and the evaluated parameters shown in Table 2. The green markers denote the two saddle points on the free energy surface and the red solid lines are the MFEPs.

The nucleation mechanism and rate are analyzed up to this point at a single supersaturation of *S* = 4.01, which was shown to be the point at which a spinodal decomposition occurs (25). To evaluate the effect of increased supersaturation on the mechanism, we redid the free energy calculations at a concentration of *m* = 18.0 mol/kg, which corresponds to a supersaturation of *S* = 4.8. The resulting free energy surface is shown in Fig. 4(a) along with the MFEP. In comparison to the free energy surface obtained at the lower supersaturation, the MFEP is further away from the *n_c_* = *n*_ρ_ line, which indicates that there is a thicker layer of amorphous particles around the crystalline core. Going from mild to severe supersaturation, the mechanism moves away from the classical pathway.

Looking at the free energy pr ofile along the MFEP (Fig. 4b), it is apparent that at *m* = 18.0 mol/kg, there are two distinct free energy barriers that the system needs to cross for nucleation. This agrees with the definition of the two-step process (51,52), where two barriers must be overcome. At *m* = 15.0 mol/kg, however, a single barrier is visible, indicating that a shift in mechanism occurs between the two concentrations. The composite cluster-free energy model given in Eq. 22 was fit onto the free energy surface obtained at *m* = 18.0 mol/kg and the thermodynamic parameters were obtained from parameter estimation, which are listed in Table 2. One of these parameters, μ_*AS*_, was estimated as 3.45 ± 0.25 kJ/mol_ion_. The positive value of this parameter suggests that the amorphous phase is now more stable than the solution phase, as opposed to the lower supersaturation case, where the amorphous phase was the least stable. The change in the sign of μ_*AS*_ further confirms the shift in mechanism going from *m* = 15 to *m* = 18 mol/kg. To investigate the change in the mechanism further, we used the evaluated thermodynamic parameters in Eq. 22 and extrapolated the free energy surface to an extended collective variable space, as shown in Fig. 4(c). The extrapolation reveals the presence of a second saddle point at *n*_ρ_ = 114 and *n_c_* = 1, denoted with a green marker on Fig. 4(c). At this supersaturation, there are now two possible pathways the system can follow as it nucleates. The first one is the pathway that is already visible on Fig. 4(a), where *n*_ρ_ and *n_c_* increase simultaneously after the formation of amorphous clusters of about 40 ions. The second pathway, which is not visible in the free energy surface obtained from simulations, goes through the formation of large metastable amorphous clusters (*n*_ρ_ > 200), after which *n_c_* starts to increase, meaning that the crystalline cluster starts to form within the amorphous cluster. The second pathway appears because the amorphous phase is now more stable compared to the solution phase, hence it is thermodynamically feasible for large amorphous clusters to form. The free energy penalties for the two MFEPs differ by 1.5 kT, with a slight thermodynamic favor for the pathway through formation of large amorphous clusters. Kinetic favor for amorphous phase nucleation was reported earlier for nucleation in undercooled liquids using density-functional simulations (57). Experimentally, Tan et al. (58) observed a decoupling between change in density and change in crystalline order in liquid to solid transition of colloids. Experimental studies of mineral nucleation report the formation of amorphous prenucleation clusters in both calcium phosphate (59) and calcium carbonate (60,61) nucleation. Using in situ TEM imaging of CaCO_3_ nucleation, Nielsen et al. (60) observed multiple pathways of nucleation at varying supersaturation conditions. Our study shows that the change in mechanism could be due to a change in the stability of the amorphous clusters. Our findings also agree with the experimental observations for crystal nucleation of potassium dihydrogen phosphate (KDP) solution (62), where different pathways were observed depending on the level of supersaturation. Similar results were observed in previous computational studies of model systems. For example, a study of crystallization from solution using a Potts lattice gas model (63) showed that the preferred nucleation pathway on the cluster size—crystallinity space changed as the nucleation temperature was altered, caused by a change in relative chemical potentials of the liquid and solid phases due to the shift in temperature. The seminal paper by Ten Wolde and Frenkel (64) reported a change in nucleation mechanism from one-step to two-step as the system gets close to the liquid–liquid coexistence point for colloids with short-range attraction. Addula and Punnathanam (65) studied the two-step mechanism in crystal nucleation from Lennard–Jones vapor and reported a very similar free energy surface to Fig. 4(c), even though they examined a low driving force region of the phase diagram, at which their computed nucleation rates were virtually zero. They studied two pressures that were above the liquid–vapor coexistence curve. It could be expected that, similar to the results of this study, a further decrease in pressure into the region where the liquid phase is no longer stable would result in the disappearance of the liquid phase saddle point. A change in the free energy surface similar to the one observed in this study, depending on the relative stability of the intermediate phase compared to the mother phase was also observed for a 2D lattice model (66,67). For more realistic systems, seeing the second saddle point from all-atomic free energy calculations is challenging, since much larger box sizes would be required to avoid finite size effects. Although model extrapolation should be handled with caution, it gives us valuable insight about the change in the mechanism which would have been challenging to get otherwise.

## Conclusions

In this paper, we examined the mechanism of NaCl nucleation from aqueous solution at elevated concentrations by providing a complete thermodynamic picture through MD simulations and free energy calculations. We showed that at *S* = 4.01, there is a thermodynamic preference for nucleation through the formation of clusters that have a layer of amorphous particles around the crystalline core, referred to as composite clusters. However, the free energy profile along the MFEP showed a single barrier, which indicates that nucleation occurs through a single-step mechanism, even though the mechanism is nonclassical due to the composite structure of the clusters. A free energy model that considers the formation of an intermediate phase was successfully fit against the mapped free energy profile, allowing for evaluation of thermodynamic parameters like the chemical potential differences between solution, amorphous, and crystalline phases. It was concluded that at this supersaturation, the amorphous phase is less stable than the solution phase, which explains the single-step behavior seen at *S* = 4.01. The nucleation rate was also computed at this supersaturation by constructing a 2D mesoscopic system. The resulting rate agrees well with the literature value obtained using CNT, which is explained by the single-step mechanism and the narrow cluster size distribution along the transition curve. As the supersaturation is further increased to *S* = 4.8, it was observed that the MFEP moves further away from the classical pathway. The chemical potential difference between the amorphous phase and the solution phase, obtained from the free energy model fit, revealed that the amorphous phase is now more stable than the solution phase allowing for large amorphous clusters to form in solution. The change in the relative stability of the amorphous phase resulted in the emergence of a second saddle point on the free energy surface, along with a second possible pathway for nucleation. This pathway involves formation of the amorphous clusters composed of more than 200 ions, after which the transition to a crystalline cluster takes place in a second step, in agreement with the two-step pathway.

Through a complete mapping of free energy surfaces with phase specific nucleus size coordinates at two different points on the phase diagram, we were able to demonstrate how supersaturation can change the stability ranking of the solution, amorphous and crystalline phases. The shift in the stability ranking was reflected onto the shape of the free energy surface and the preferred pathways for nucleation. Finally, we showed that the composite cluster-free energy model can effectively describe the thermodynamics not only in simple model systems (67), but also in a realistic system for solute precipitate nucleation. We believe that the methodology used in this paper can be extended to polymorphic systems to reveal thermodynamic as well as kinetic insight into competing polymorphs.

## Funding

Financial support for this work was provided by AbbVie Inc. through grant numbers 8000053025 and 8000069224.

## Supplementary Material

pgac033_Supplemental_Files

## Data Availability

The data that support the findings of this study (umbrella sampling data at *m* = 15 and *m* = 18 mol/kg and trajectory data for diffusivity calculation at *m* = 15 mol/kg) are available for download from the Open Science Foundation Data Repository with DOI: 10.17605/OSF.IO/MJGEP.
