# Supplementary material for: An investigation of the kinetics and thermodynamics of NaCl nucleation through composite clusters
Source: PNAS Nexus. 2022 Mar 30;1(2):pgac033. doi: 10.1093/pnasnexus/pgac033 (PMC9802385; doi:10.1093/pnasnexus/pgac033)
Supplement: pgac033_Supplemental_Files [file pgac033_supplemental_files.zip › PNASNEXUS-PNASNEXUS-2021-00267-s01.pdf]

Supplementary Information: An investigation of the kinetics and thermodynamics of NaCl nucleation through composite clusters

Pelin S. Bulutoglu[1], Shiyan Wang[1], Moussa Boukerche[2], Nandkishor K. Nere[2], David S. Corti[1], and Doraiswami Ramkrishna[1]

[1]Davidson School of Chemical Engineering, Purdue University, West Lafayette, IN. 47907-2100, USA.
[2]Process Research and Development, AbbVie Inc, North Chicago, IL

March 3, 2022

1 HMC/MD Simulations

HMC/MD simulations are done with LAMMPS by using a python wrapper script that drives the MD simulator for the MD trajectories and performs the velocity initialization, CV and bias potential evaluation and acceptance check steps. Accuracy of the pppm solver is raised to 10^{-6} in the HMC/MD simulations to prevent energy drift, which can adversely affect detailed balance. To ensure time reversibility of the MD trajectories in each step, the water molecules are simulated as rigid groups instead of using the numerically solved RATTLE algorithm to constrain the hydrogen bonds. Each Monte Carlo step consists of a 2 ps long microcanonical MD trajectory followed by an acceptance check with an acceptance probability based on the Metropolis criterion

$$\mathcal{P}_{acc} = \min\left(1, \exp\{-\beta(\Delta PE + \Delta KE + \Delta U_B)\}\right), \tag{1}$$

where ΔPE, ΔKE and ΔU_B are the changes in potential energy, kinetic energy and biasing potential, respectively.

2 Free energy calculation and validation

We obtained the free energy profile as a function of the crystalline cluster size, n_c, at a concentration of 15.0 mol/kg to compare with the results of Jiang *et al.* [2] As shown in Fig. S1, there is good agreement between our results and the literature, validating our simulation methods.

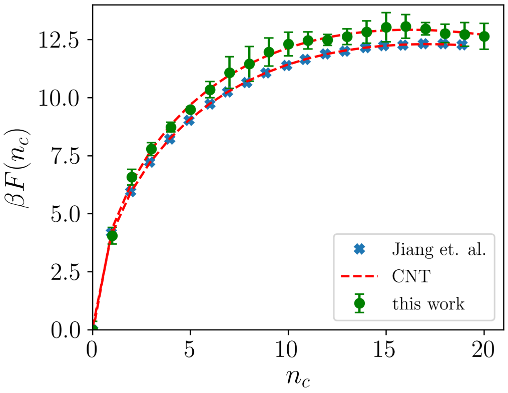

Figure S1: Free energy of formation of a crystalline cluster of size n_c at solution concentration of 15 mol/kg. The blue crosses are the data points taken from Jiang *et al.* [2], green circles are the data from this work, obtained from hybrid MC/MD simulations with umbrella sampling. The red dashed lines are fits of CNT into the data.

The order parameters used in this study are the sizes of the largest dense (n_ρ) and crystalline (n_c) clusters in the simulations box. As such, the free energy surfaces reported in this paper are based on logarithm of the probabilities of any cluster size being the largest in the system. For small cluster sizes, this definition of free energy does not agree with CNT which is related to the probability that a given cluster has size n [1, 4]. To demonstrate the difference between two free energy profiles on a single order parameter, we obtained two distributions [3]: $P(n_c)$, which is the probability that the largest cluster obtained from an equilibrium configuration is n_c, and $N(n_c)$, which is the average number of clusters with size n_c in an equilibrium configuration. $N(n_c)$ was estimated by counting all clusters in sample configurations from an unbiased MD simulation. For example, in a configuration where the largest cluster size is 3, $N(0) = 974$ (number of aqueous ions,) $N(1) = 18$ (number of ions that are "solid-like",) and $N(2) = 1$. Figure S2 shows the 1D free energy profiles obtained by using $P(n_c)$ for all nucleus sizes as opposed to using $N(n_c)$ for small cluster sizes and $P(n_c)$ for larger cluster sizes at m=15 mol/kg. The two profiles converged for $n_c > 6$ at this supersaturation. It is visible that

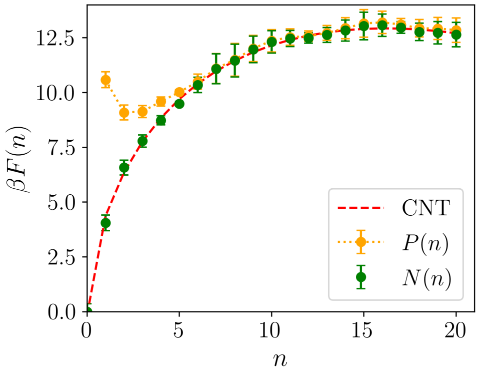

Figure S2: The difference between free energy profiles when $P(n_c)$ is used for all cluster sizes (yellow markers) vs. when the $N(n_c)$ correction is made for small cluster sizes (green markers). Red dashed line shows the CNT fit onto the corrected profile.

the $P(n_c)$ based profile diverges as n_c goes to values smaller than the average largest cluster in the simulation box. CNT expression agrees well with the free energy result when the $N(n_c)$ correction is made for small cluster sizes.

For calculating the 2D free energy surface, we made a similar correction of the nucleus size distribution for small nucleus sizes. For $n_c < 6$ and $n_\rho < 20$, we counted all clusters in configurations obtained from an unbiased simulation for 50 ns and recorded the positions of their centers and their radii. If the distance between the centers of a crystalline and dense cluster was shorter than the radius of the dense clusters, it was assumed that the dense cluster encapsulated the crystalline clusters, hence, $n_c > 0$ and $n_\rho > 0$. Else, it was assumed that the dense cluster did not contain any crystalline particles, hence $n_c = 0, n_\rho > 0$. The histogram of small cluster sizes thus obtained was used in WHAM along with the histograms obtained from umbrella sampling simulations to estimate the full 2D free energy surface.

3 Sensitivity of results to choice of order parameter

The choice of cut-off values when calculating order parameters, such as the number density cut-off value for detecting solid-like particles or the distance criterion for detecting neighbors, can result in over/underestimation of the cluster sizes. Here we conduct an analysis to understand the effect of one of those criteria, the number density cut-off value, on the main conclusions of this paper. The number density cut-off value, N_{cut}, is chosen as 8, such that the ions for which $\rho > 8$ are marked as "solid-like" and are subject to the clustering algorithm to find the largest dense cluster in the system. The choice of the cut-off value is based on the distribution of number density of ions in a system with a crystalline slab embedded in aqueous solution, shown in Fig. S3. There are three distinct peaks in the distribution: the largest peak corresponding to the ions that are inside the crystal, the middle peak corresponding to the surface ions and the lowest peak corresponding to the ions in solution. $\rho = 8$ falls between the solution and surface ion peaks to ensure that the ions that are on the surface of clusters are still being counted as solid-like. The size of the detected dense clusters, however, depends strongly on the chosen cut-off, as shown in Fig. S4. By fitting a power curve to the average dense nucleus size vs. N_{cut} data, we calculate that choosing $N_{cut} = 7$ would result in dense clusters roughly 1.6 times that obtained with $N_{cut} = 8$. On the other hand, choosing a larger value for the cut-off, $N_{cut} = 9$, would result in dense clusters roughly 0.7 times that of those obtained in this study. For standard free energy calculations, if an order parameter consistently overestimates or underestimates the cluster size, this will not affect the resulting free energy barrier, but it will affect the *location* of the barrier [5]. So, the resulting location of the saddle point from our 2D free energy calculations, as well as the parameters obtained from the composite cluster model fit can be affected by our choice of order

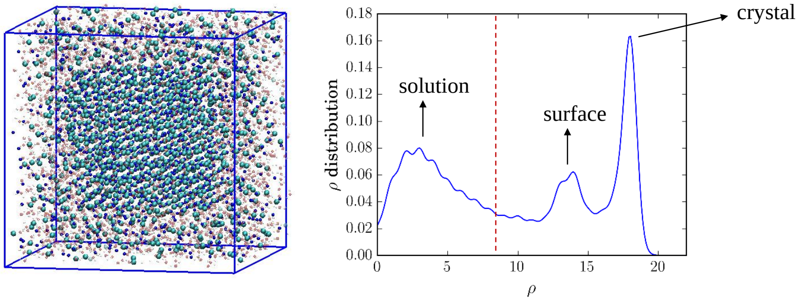

Figure S3: Distribution of number density of ions in a system with a crystalline slab embedded in solution as shown on the left.

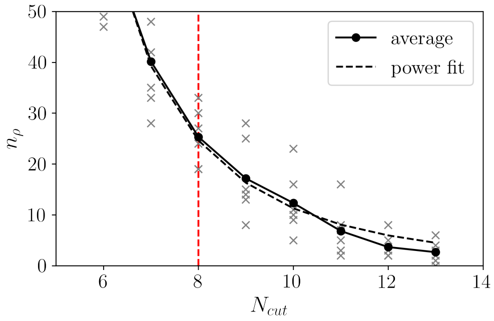

Figure S4: Largest cluster size detected in a configuration as N_{cut}, which is the number density cutoff such that an ion is deemed "solid-like" if $\rho > N_{cut}$, is increased. The filled circles are the averages of data from 5 individual configurations, shown in grey crosses. The dashed line is a power curve fit on the average curve.

parameter. To see the effect of the change in the cut-off value, the dense cluster sizes were multiplied by factors of 1.6 and 0.7, and the parameters from the composite cluster fit were re-evaluated. The resulting free energy surfaces and composite cluster model fits are shown in Fig. S5.

The values of μ_{AS} obtained from the fits are -0.51, -0.63 and -0.91 kJ/mol$_{\text{ion}}$ for the free energy surfaces obtained with N_{cut} values of 9, 8, and 7, respectively. The value is negative, regardless of the cut-off value, showing that the qualitative conclusion about the amorphous phase stability is not sensitive to the order parameter. It should be noted, however, that this is a rough calculation and a true sensitivity analysis of the fit parameters to the OPs is not possible without performing the free energy calculations with changing values of the OP criteria.

4 Cluster shape and structure analysis

The composite cluster model is an extension to CNT, and the underlying assumptions of CNT such as the capillarity approximation and spherical cluster assumption are used in the composite cluster model as well. Since this study focuses on high supersaturations, the critical cluster sizes are quite small (consisting of about 35 ions at m=15 and about 50 ions at m=18 mol/kg). At these small sizes, it is unrealistic to expect perfectly spherical clusters. A sphericity analysis was done by computing the moment of inertia tensor of the clusters sampled along the MFEP at both concentrations and calculating $S = \lambda_{min}/\lambda_{max}$, where λ_{min} and λ_{max} are the smallest and largest eigenvalues of the moment of inertia tensor, respectively. S will be close to 1 if the cluster is perfectly spherical and close to zero if it is far from spherical. Fig. S6 shows the average S values of 80 configurations with similarly sized clusters at different points along the MFEP both at m=15 mol/kg and m=18

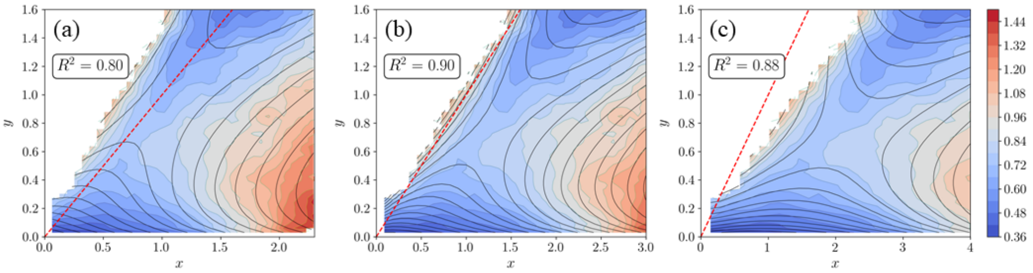

Figure S5: Free energy surfaces and fits of the composite cluster model when the dense cluster size is consistently (a) underestimated by a factor 0.7, (b) unchanged, (c) overestimated by a factor of 1.6 compared to the cluster sizes computed with the density cut-off of 8.

mol/kg. The clusters are never fully spherical at the cluster sizes seen along the MFEP. As cluster

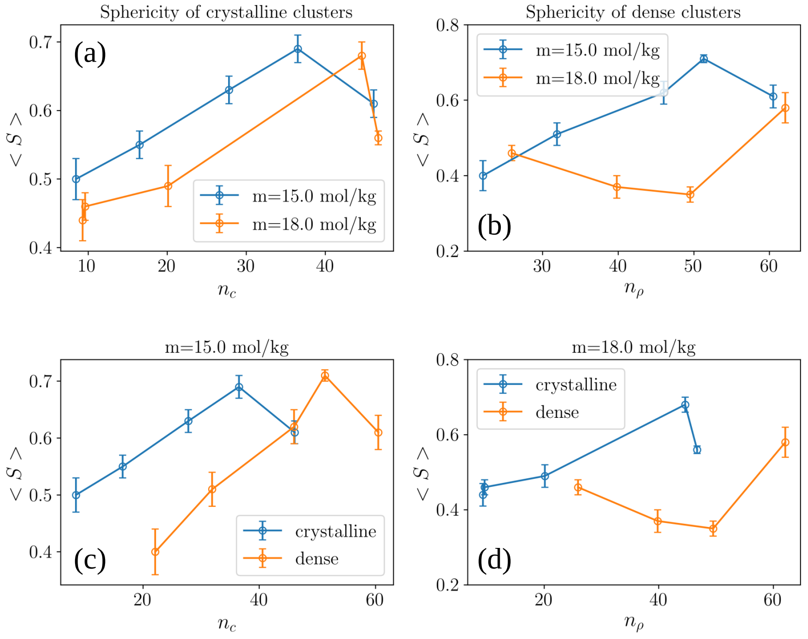

Figure S6: Average sphericity values of clusters obtained from increasing cluster size configurations along the MFEP.

size increases, the clusters tend to get slightly more spherical. There is also a statistically significant decrease in sphericity of clusters when the supersaturation is increased, which can be observed in Fig. S6 (a) and (b). Fig. S6 (c) and (d) shows that crystalline clusters are generally more spherical than the dense clusters of the same size at both supersaturations, due to the cubic shape of the unit cell of crystalline NaCl.

Another important point is the change in the structure of the nucleus as it is going through stages from the precritical size to nucleation and growth. The percentage of crystalline ions in the cluster is plotted against the total number of ions in the cluster at both supersaturations in Fig. S7. At $m = 18$ mol/kg, the pathway going through the second saddle point detected from composite cluster model extrapolation is also included in Fig. S7. It should be noted that the cluster sizes on this pathway are derived from the MFEP obtained from the model fit. A continous increase in crystallinity is observed as the cluster grows along the MFEP at $m = 15$ mol/kg. At the lower supersaturation, the mechanism resembles a classical pathway the most and the cluster is mostly crystalline both at the critical size (marked with green filled circle) and beyond. It is seen that the cluster includes around 10

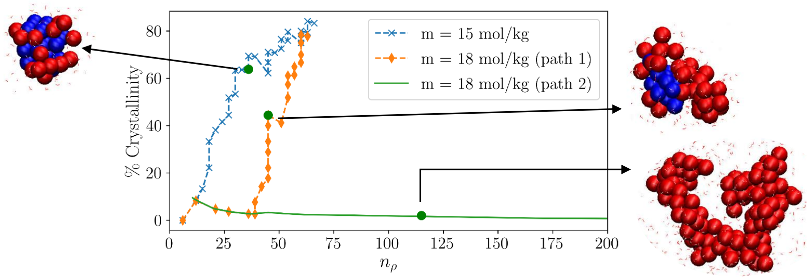

Figure S7: The pathways of nucleation plotted in terms of the percentage of crystalline ions in the cluster and the dense cluster size. The transition state along each pathway is denoted with a green filled circle. Snapshots of clusters close to the saddle points are also shown, with amorphous ions shown in red and crystalline ions shown in blue.

amorphous ions and the number of amorphous ions stays mostly constant as the cluster grows along the MFEP. At $m = 18$ mol/kg, we see two pathways: Along the first pathway, the cluster starts off as amorphous until it reaches a size of about 45 ions, after which the crystallinity of the cluster starts increasing. The saddle point is reached only when the crystallinity increases to about 40%, which then continues to increase as the cluster grows. Along the second pathway, which was not present at the low supersaturation case, the crystallinity stays at essentially zero as the saddle point is traversed and the cluster enters the growth stage.

References

[1] Vishal Agarwal and Baron Peters. Solute precipitate nucleation: A review of theory and simulation advances. *Advances in Chemical Physics: Volume 155*, pages 97–160, 2014.

[2] Hao Jiang, Pablo G Debenedetti, and Athanassios Z Panagiotopoulos. Nucleation in aqueous nacl solutions shifts from 1-step to 2-step mechanism on crossing the spinodal. *The Journal of chemical physics*, 150(12):124502, 2019.

[3] Sarah EM Lundrigan and Ivan Saika-Voivod. Test of classical nucleation theory and mean first-passage time formalism on crystallization in the lennard-jones liquid. *The Journal of Chemical Physics*, 131(10):104503, 2009.

[4] Lutz Maibaum. Comment on "elucidating the mechanism of nucleation near the gas-liquid spinodal". *Physical review letters*, 101(1):019601, 2008.

[5] Nils ER Zimmermann, Bart Vorselaars, Jorge R Espinosa, David Quigley, William R Smith, Eduardo Sanz, Carlos Vega, and Baron Peters. Nacl nucleation from brine in seeded simulations: Sources of uncertainty in rate estimates. *The Journal of chemical physics*, 148(22):222838, 2018.
